# Rheological Performance Analysis of Different Preventive Maintenance Materials in Porous High-Viscosity Asphalt Pavements

**DOI:** 10.3390/ma17071458

**Published:** 2024-03-22

**Authors:** Bin Xu, Weiying Wang, Yiren Sun, Mingyang Gong

**Affiliations:** 1 Research and Development Center of Transport Industry of New Materials, Technologies Application for Highway Construction and Maintenance, Zhong Lu Gao Ke (Beijing) Road Technology Co., Ltd., Ministry of Transport, Beijing 100088, China; xubin8191@126.com; 2 The Key Laboratory of Road and Traffic Engineering, Ministry of Education, Tongji University, 4800 Cao’an Road, Shanghai 201804, China; 3 School of Transportation and Logistics, Dalian University of Technology, Dalian 116024, China; sunyiren@dlut.edu.cn

**Keywords:** porous asphalt pavement, preventive maintenance, rheological properties, temperature sensitivity, low-temperature crack resistance

## Abstract

Porous asphalt pavements are widely used in rainy and wet areas for their skid resistance, noise reduction, runoff minimization and environmental sustainability. Long-term moisture vapor erosion and the destabilization of large pore structures can easily result in pavement problems such as fragmentation, spalling, cracking, and excessive permanent deformation. To this end, four different preventive maintenance materials, including the rejuvenation (RJ), cohesion reinforcement (CEM), polymerization reaction, and emulsified asphalt (EA) types, were selected in this paper to improve the high-viscosity porous asphalt pavement. The effects of the different preventive maintenance materials on the temperature sensitivity, rheological properties and fatigue performance of high-viscosity modified asphalt were evaluated through temperature sweep, frequency sweep, multi-stress creep recovery (MSCR), linear amplitude sweep (LAS), and bending beam rheometer (BBR) tests. The results showed that the four preventive maintenance materials exhibit different enhancement mechanisms and effects. RJ improves the fatigue properties, deformation resistance and low-temperature cracking resistance of aged asphalt by adding elastomeric components; CEM materials are more conducive to increasing the low-temperature crack resistance of aged asphalt; while GL1 and EA improve the viscoelastic behavior of aged asphalt, but the effect of the dosing ratio needs to be considered.

## 1. Introduction

Porous asphalt pavements are extensively applied in wet and rainy zones because of their benefits, such as improved drainage, reduced runoff, enhanced skid resistance, noise reduction, extended pavement life and environmental sustainability [1,2]. However, porous pavements are highly susceptible to environmental factors, especially humidity and temperature conditions [3,4,5,6]. Under repetitive loading and water vapor, pavements often suffer from aggregate raveling, adhesion failure, pavement cracking and permanent deformation, which seriously affect the pavement service life and driving safety [7,8,9,10]. Therefore, preventive maintenance techniques for porous asphalt pavements play a crucial role in improving pavement functionality and durability.

Asphalt drainage pavement is mainly composed of large voids and open-graded asphalt mixture, thus forming a spatial skeleton-void structure, which can provide greater compressive strength [11,12,13]. Due to the suspension of open-graded aggregates and the small contact position between the aggregates, ordinary asphalt struggles to provide an effective bonding effect, therefore requiring a high-viscosity modifier to increase the asphalt-bonding effect as well as the overall strength of the pavement [9,14]. However, the high void fraction results in the drainage pavement being susceptible to the external environment, such as air intrusion and rainwater washout. As a result, high-viscosity asphalt binders are highly susceptible to aging reactions with oxygen and moisture erosion, which can reduce asphalt viscosity and lead to early pavement collapse [15,16,17]. Currently, there are several solutions to this problem, such as renovation, pavement thickening and preventive maintenance. Compared to the other methods, preventive maintenance is not only resource-efficient but also more direct and effective.

The preventive maintenance method mainly utilizes preventive treatments to delay the drainage pavement damage, extend its service life, and maintain or improve the anti-skid, drainage, anti-glare and noise reduction requirements without increasing the structural load-bearing capacity [18,19]. Maintenance materials are critical to improve the effectiveness of preventive maintenance, which are mainly to added with special ingredients through the void penetration dispersed in the structural layer to restore the aging properties of asphalt or heal asphalt cracks [20,21]. To prevent cohesive damage within the drainage pavement and delay the deterioration of high-viscosity asphalt, preventive maintenance materials need to provide the following properties: (1) significant age-reducing ability; (2) compatibility with high-viscosity asphalt; (3) spreading and flowing easily in the pavement; (4) effective viscosity enhancement; and (5) fast curing. At present, there are two mainstream pre-treatment maintenance materials, including the rejuvenation and bonded types. The rejuvenation category mainly consists of oil-based rejuvenators, which increase the lightweight component of asphalt based on the blending theory, thereby slowing down the high-viscosity asphalt deterioration and reducing the aggregate loosening. The bonded materials are usually made with emulsified asphalt, binder-reinforcing agents, kaolin maps, and special activators for polymers, which increase both the cohesion and the bonding effect between the asphalt and the aggregate. For example, Lommerts et al. [22] developed a multifunctional emulsifier to treat pavements, which rapidly breaks up the emulsion under air circulation, encapsulates the aggregate surface, and repairs and enhances the inter-aggregate adhesion. Zhang et al. [23] pre-maintained large pore asphalt mixtures using a reducing agent and found that the reducing agent improved the road performance of the asphalt mixtures. Lin et al. [24] investigated the effect of reduced-type sealer materials on hot-mix asphalt mixtures and found that they have excellent permeability and value-added performance.

To gain further insight into the effectiveness of preventive maintenance materials in drainage pavements, some researchers have carried out multi-directional performance studies on preventive materials. Lee et al. [25] tested the friction, penetration capacity, mechanical properties and contact angle of soft asphalt rejuvenators, and they evaluated the influencing factors and evaluation criteria of the rejuvenators from the macro performance perspective. Karlsson et al. [26] used Fourier infrared spectroscopy to analyze the factors affecting the reductant diffusion in large-porosity asphalt mixtures based on the chemical perspective. According to the Gel Permeation Chromatography (GPC) method, Shen et al. [27] analyzed the molecular structure of aged asphalt during regeneration and evaluated the effects of different reductant concentrations. Although the above scholars have summarized the macroscopic pavement properties of preventive maintenance materials and provided some data support for the selection of maintenance materials, few studies have comprehensively evaluated the mechanical properties of different maintenance materials. Therefore, the actual mechanism of action of preventive maintenance materials in asphalt remains unclear.

To this end, four preventive maintenance materials were selected in this study to improve high-viscosity asphalt pavements, including rejuvenation (RJ), cohesion reinforcement (CEM), polymerization reaction, and emulsified asphalt (EA). Then, the temperature sensitivity, rheological properties and fatigue performance of the four preventive maintenance materials were evaluated through temperature scanning (TS), frequency scanning (FS), multi-stress creep recovery (MSCR), linear amplitude scanning (LAS) and bending beam rheometer (BBR) tests. The research results provide some technical support for the selection of preventive maintenance materials.

## 2. Materials and Specimen Fabrication Process

### 2.1. Materials

In this study, PG 58-22 (Pen 80/100) virgin asphalt binder and high-viscosity additive were utilized to fabricate the high-viscosity asphalt binder, and the properties of the two materials are shown in Table 1 and Table 2.

Four preventive maintenance materials were selected to treat high-viscosity modified asphalt, including rejuvenation (RJ) material, cohesion reinforcement (CEM) material, polymerization reaction material, and emulsified asphalt (EA). The RJ material consisted mainly of oily components that serve to restore the properties of aged asphalt; the CEM materials were prepared from an asphalt base and micron-sized kaolin, which are primarily used to improve the skid resistance of pavements; the polymerization reaction materials (GL1) were mainly composed of α-cyanoacrylate resin-based materials; and the emulsified asphalt was a commonly used preventive maintenance material.

### 2.2. Specimen Fabrication Process

The preparation process for the high-viscosity modified asphalt binder involved several steps. Initially, a high-viscosity modifier was introduced to the virgin asphalt and subjected to low-speed mixing for 15 min at 135 °C. Subsequently, the mixture was further mixed at 155 °C for 30 min to ensure thorough blending. By following these steps, a warm mix of recycled asphalt can be obtained.

In this study, laboratory aging was used to obtain an artificial RAP binder. During the aging process, continuous short-term rolled thin-film oven (RTFO) aging [28] was preferred for the matrix binder, followed by long-term pressurized aging of the vessel [29]. In addition, the preventive maintenance materials were solvent-based liquid materials, which can be added directly to the high-temperature molten aging asphalt for adequate mixing. The mixing temperature was 140 °C and the contents of the four preventive maintenance materials were all chosen as 5%, 10% and 15%.

## 3. Test Methods

### 3.1. Temperature Sweep (TS) Test

Temperature sensitivity is an important indicator affecting the properties of asphalt. In this paper, the TS test was conducted using a dynamic shear rheometer (DSR) to investigate the temperature sensitivity of the different asphalt binders, in which the loading time was 0.5 h, the loading frequency was 10 rad/s, the stress level was 100 Pa, and the temperature scanning range was 30–80 °C. The temperature rise mode was linear, once every 140 s, and 5 °C each time.

### 3.2. Multi-Stress Creep Recovery (MSCR) Test

In the MSCR test, two stress levels were used for continuous testing (0.1 kPa and 3.2 kPa) [30]. Ten cycles were performed for each stress level, each cycle consisting of a one-second creep phase and a nine-second unloaded recovery phase, for a total test time of two hundred s. The test temperature was 46~64 °C. Finally, the irrecoverable creep compliance *J_nr_*_3.2_ and the recovery rate R were considered as the evaluation indexes, in which the irrecoverable creep compliance served as a high-temperature evaluation index for characterizing the rutting resistance of the asphalt binder, while the creep recovery rate reflected the composite deformation after ten creep recovery cycles at each stress level. The *J_nr_*, *R* and *J_nr-diff_* are calculated as follows:

(1) $$\%R(\tau )=\frac{\sum_{i=1}^{10}{\left(\frac{e_{1}-e_{0}}{e_{1}}\right)}_{i}}{10}\times 100$$

(2) $$J_{nr}(\tau )=\frac{\sum_{i=1}^{10}{\left(\frac{e_{10}}{\tau }\right)}_{i}}{10}$$

(3) $$J_{\text{nr-diff}}=\frac{J_{nr}(3.2\mathrm{kPa})-J_{nr}(0.1\mathrm{kPa})}{J_{nr}(0.1\mathrm{kPa})}$$

where *R* is the creep recovery rate; *J_nr_* is the non-recoverable creep compliance, kPa^−1^; *e*_1_ is the strain at the end of the loading cycle; *e*_10_ is the residual strain at the end of the corresponding recovery cycle; *τ* is the shear stress; and *J_nr-diff_* is the unrecoverable creep compliance difference.

### 3.3. Frequency Sweep Test

The frequency sweep test was conducted in strain-controlled mode to evaluate the linear viscoelastic behavior of the asphalt binders at a given combination of temperature and frequency. The frequency range used in this study spanned from 0.1 Hz to 50 Hz, while the temperature range covered 4 °C to 76 °C, with intervals of 12 °C. A consistent load was applied for a duration of 15 min at each temperature. The relationship between the components of the complex modulus G* is shown below [31,32]:

(4) $$G^{*}=G^{\prime }+iG^{\prime \prime }=\left|G^{*}\right|\cos\delta +i\left|G^{*}\right|\sin\delta$$

where *G*′ is the storage modulus; *G*″ is the loss modulus; |G*| is the dynamic modulus; and *δ* is phase angle. The complex moduli of the different frequencies and temperatures can be shifted along the frequency axis on a logarithmic scale to form a master curve at a predetermined reference temperature. The displacement factor *a_T_* is expressed as follows:

(5) $$t_{r}=\frac{t}{\alpha_{T}}$$

(6) $$\omega_{r}=\omega \times \alpha_{T}$$

where *ω*_r_ is the reduced angular frequency; and *ω* is the angular frequency.

To ensure the model’s accuracy, the Christensen–Anderson–Marasteanu (CAM) model was chosen to fit the viscoelastic parameters of asphalt. The CAM model expression is as follows:

(7) $$G^{*}=\frac{G_{g}^{*}}{{\left[1+{\left(f_{c}/f_{r}\right)}^{k}\right]}^{m/k}}$$

where G^*^ is the complex shear modulus; $G_{g}^{*}$ is the glassy modulus; *f_r_* and *f*_c_ are the reduced frequency and crossover frequency; and *k* and *m* are the model parameters.

### 3.4. Accelerated Fatigue Test

The linear amplitude sweep (LAS) test was used to evaluate the asphalt fatigue performance according to AASHTO TP 101 [33]. The sinusoidal wave load was used for loading, and the amplitude was increased linearly from 0.1% to 30%. In addition, the scan time was 5 min. The test was conducted using a dynamic shear rheometer with a test temperature of 20 °C and a loading time of 20 min.

### 3.5. Low-Temperature Bending Creep Test

In this study, the BBR test was carried out to evaluate the low-temperature performance (LTP) of the different preventive maintenance materials by analyzing the stress relaxation characteristics of asphalt at low-temperature constant load pressure according to AASHTO T313-12 [34]. Two evaluation indexes, the creep stiffness modulus S and creep rate m, were adopted to evaluate the resistance to permanent deformation of asphalt and the change rate of asphalt stiffness under load, respectively. The experimental temperatures for the BBR test were −12 °C and −18 °C, and the test temperature was 30 min. Based on the displacement change of asphalt under a constant load, the creep stiffness modulus *S*(*t*) can be obtained:

(8) $$S\left(t\right)=\frac{PL^{3}}{4bh^{3}\delta (t)}$$

where *S*(*t*) is the creep stiffness modulus; *P* is the constant load; *L* is the trabecular spacing; *b* is the trabecular width; and *h* is the trabecular height. In each of the above experiments, three test specimens were used as parallel specimens and the results of the three experiments were averaged for display.

## 4. Results and Analysis

### 4.1. Temperature Sensitivity Analysis

**Figure 1:**
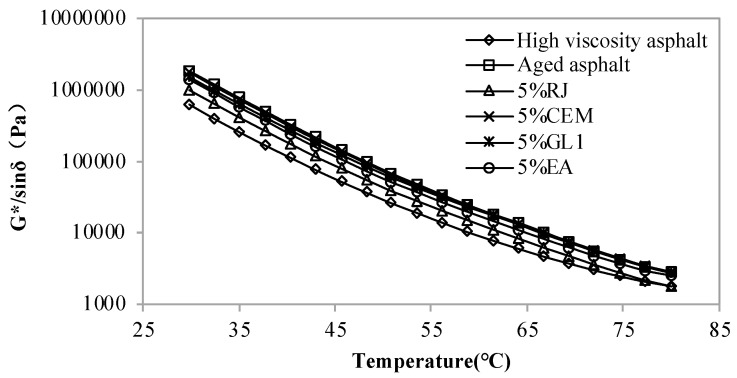
Rutting factor of new asphalt with 5% content of pre-maintenance material at different temperatures.

**Figure 2:**
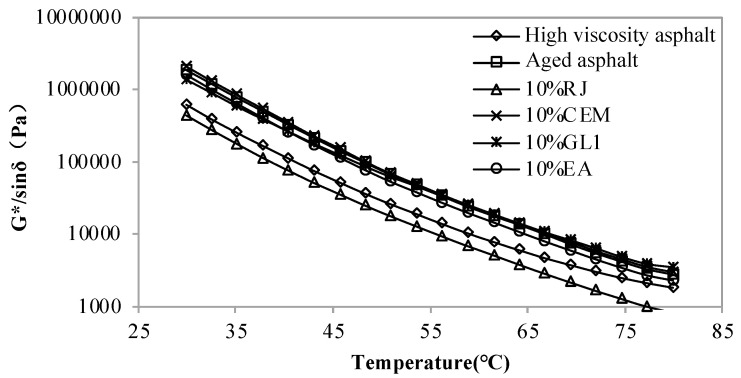
Rutting factor of new asphalt with 10% content of pre-maintenance material at different temperatures.

**Figure 3:**
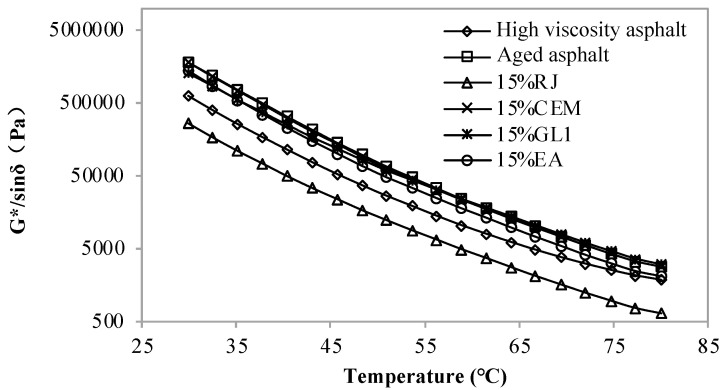
Rutting factor of new asphalt with 15% content of pre-maintenance material at different temperatures.

Figure 1, Figure 2 and Figure 3 display the rutting coefficients of new asphalt with different contents of RJ, CEM, GL1 and EA at different temperatures. As observed, the rutting coefficient of the aged asphalt was greater than that of the original one, which also indicates that the asphalt hardens and increases its resistance to deformation after aging. The RJ decreases the rutting factor of aged asphalt and the one decreases with the increasing RJ content. However, the rutting coefficients are lower than those of the original high-viscosity asphalt when the material content exceeds 10%, and the rutting resistance is obviously insufficient. Therefore, the RJ content needs to be less than 10%. Comparing the three figures, it can be seen that the rutting factor of asphalt is basically constant before and after adding CEM, and there is no significant performance difference with the changing of the CEM content, indicating that the CEM material is ineffective in relation to the temperature sensitivity of aging asphalt.

For the GL1 material, the rutting factor of the improved asphalt is slightly smaller than that of the aged one at 30–50 °C; after the temperature exceeds 50 °C, the effect of the GL1 gradually decreases and the asphalt rutting factor does not change with the GL1 material content. This is because the melting point of GL1, as a mastic polymer reactive material, is lower than that of asphalt, which provides some improvement in the low temperature range. As the temperature increases, the GL1 becomes compatible with the asphalt, thus the reclamation effect slowly disappears. Before and after the addition of the EA materials, the rutting factor of the aged asphalt was reduced to some extent, albeit to a lesser extent. This means that although the EA material has some improvement effect, the effect is not obvious.

### 4.2. MSCR Test Results

**Figure 4:**
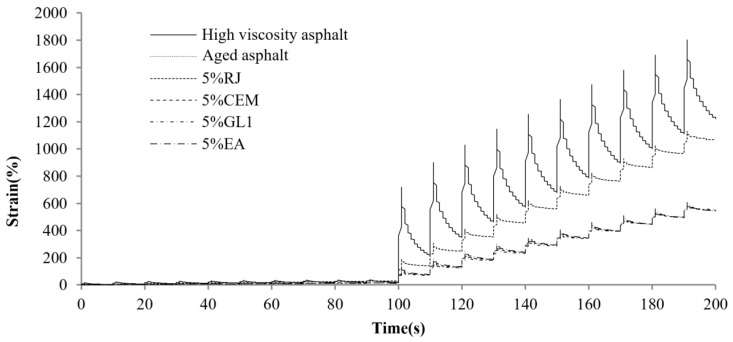
MSCR test curves of virgin high-viscosity asphalt, aged asphalt and new asphalt with 5% maintenance material content.

**Figure 5:**
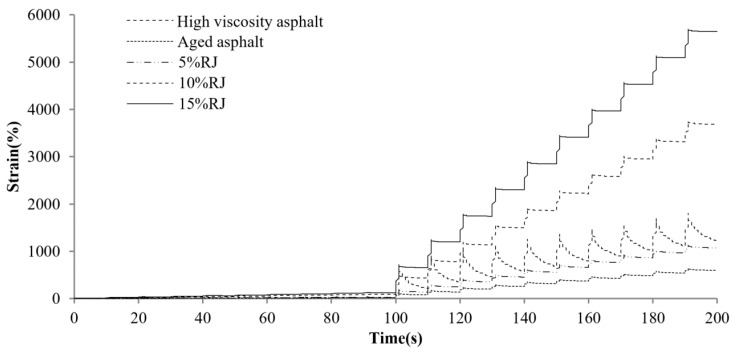
MSCR test curves of new asphalt with different RJ contents.

**Figure 6:**
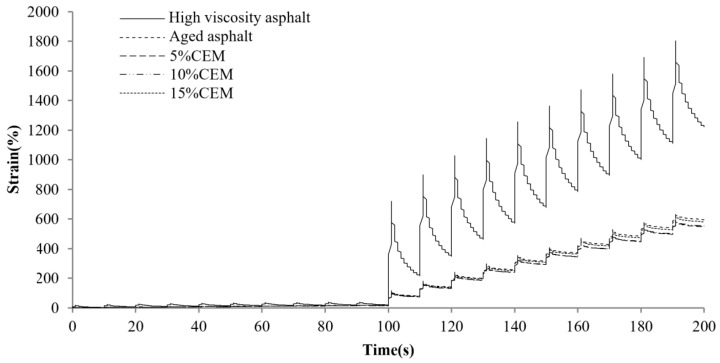
MSCR test curves of new asphalt with different CEM contents.

**Figure 7:**
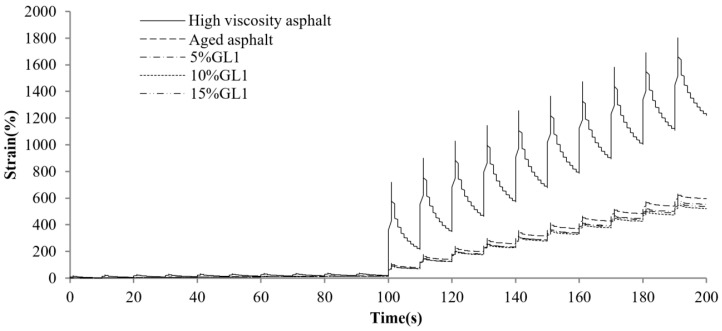
MSCR test curves of new asphalt with different GL1 contents.

**Figure 8:**
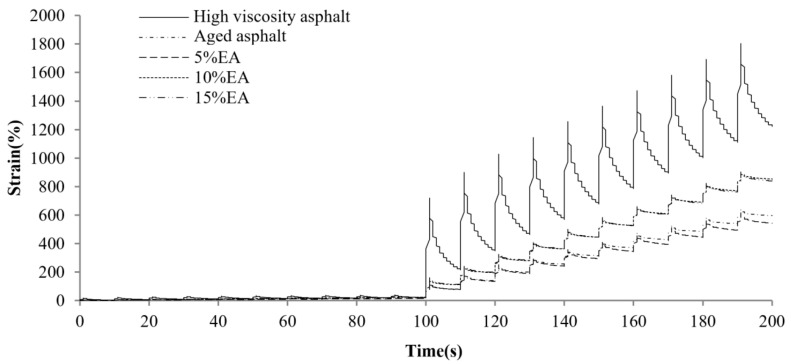
MSCR test curves of new asphalt with different EA contents.

Figure 4 displays the MSCR comparison results for different asphalts at a preconditioning material content of 5%. As observed, the total creep deformation of the original high-viscosity asphalt is the largest, and the deformation recovery is faster in the recovery phase, indicating that it provides excellent elastic recovery performance. The total creep deformation as well as the elastic deformation of high-viscosity bitumen decreases dramatically after PAV aging. This means that the asphalt elastic component weakens and gradually hardens. Along with the incorporation of different preventive maintenance materials, the creep and recovery rates of asphalt exhibited various improvements. The best effect is related to the RJ material, as the aging asphalt creep deformation significantly increased after the RJ addition and was even basically restored to the unaged state. This is related to the oily substance of the material, as RJ increases the oil content while acting as a lubricant for the asphalt, reducing its resistance to deformation. This indicates that CEM, GL1 and EA find it difficult to alter the asphalt resistance to deformation at lower contents. Figure 5, Figure 6, Figure 7 and Figure 8 present the effect of different preventive maintenance material contents on asphalt creep. It can be seen that as the content of the maintenance material increases, the improvement effect of RJ and EA gradually increases, but the effect of CEM and GL1 on the total creep is basically unchanged. Especially for RJ, the effect of the increased creep values is particularly pronounced, thus RJ materials considerably reduce the deformation resistance of asphalt binder.

**Figure 9:**
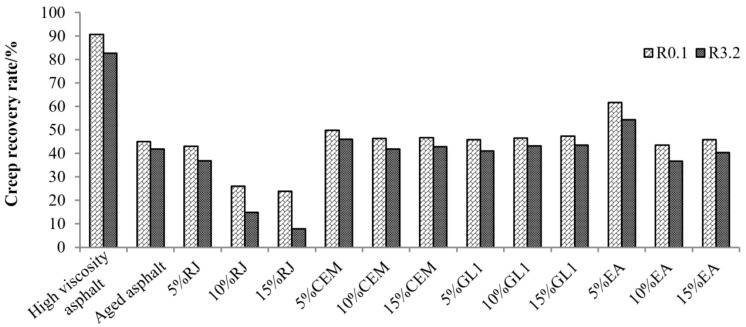
Comparison of the creep recovery rate R for asphalt at different stresses (60 °C).

**Figure 10:**
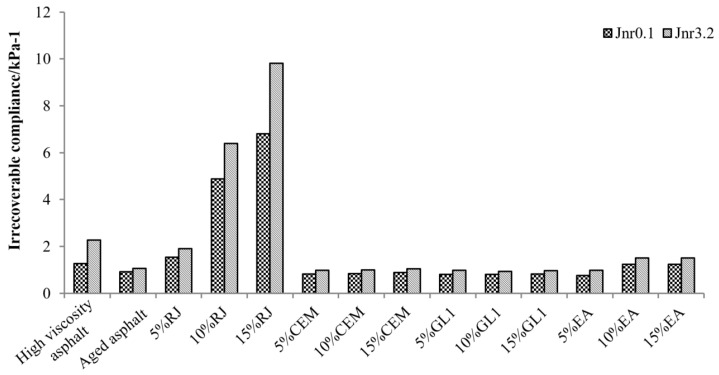
Comparison of the irrecoverable compliance *J_nr_* for asphalt under different stresses (60 °C).

According to Equations (1)–(3), the creep recovery (*R*) and compliance (*J_nr_*_3.2_) can be obtained, and the results are shown in Figure 9 and Figure 10. The *R* value characterizes the elastic deformation capacity of the asphalt. As shown in Figure 9, the creep recovery of high-viscosity asphalt is the largest, while that of aged asphalt decreases significantly by up to 50. As the content of RJ increases, the *R* value of aged asphalt gradually decreases, especially at 3200 Pa stress, when the *R* value was basically halved, indicating that the creep recovery performance of RJ was relatively weak under high-stress conditions. The *R* value of asphalt under CEM and GL1 is close to that of aged asphalt, and the material content has essentially no effect on the creep recovery. In addition, the *R* value of the new asphalt showed a fluctuating trend with the increasing of the EA content, but the amplitude was insignificant, indicating that the effect of EA on the asphalt creep recoverability was moderate.

The permanent deformation accumulation mainly depends on the magnitude of the *J_nr_* value. Figure 10 shows the results concerning the irrecoverable compliance for six asphalts at different stresses. It can be noted that asphalt exhibits higher unrecoverable elasticity at high stress levels. The irrecoverable compliance of high-viscosity asphalt decreases significantly after aging, and the resistance to permanent deformation decreases. After adding RJ material to high-viscosity aged asphalt, the irrecoverable compliance becomes larger and higher than that of aged asphalt. As the content increases, the irrecoverable flexibility increases significantly, seriously weakening the deformation resistance. However, asphalt is prone to plastic flow deformation under repeated loading and exhibits viscous fluid properties, thus it is important to control the content of RJ material. The unrecoverable compliance of GL1 and CEM with aged asphalt is basically the same, indicating that these two materials cannot improve the creep performance of aged asphalt at high temperatures. The irrecoverable compliance of EA materials increases slightly at high contents, suggesting that increasing the EA content contributes to the high-temperature performance of aged asphalt.

**Figure 11:**
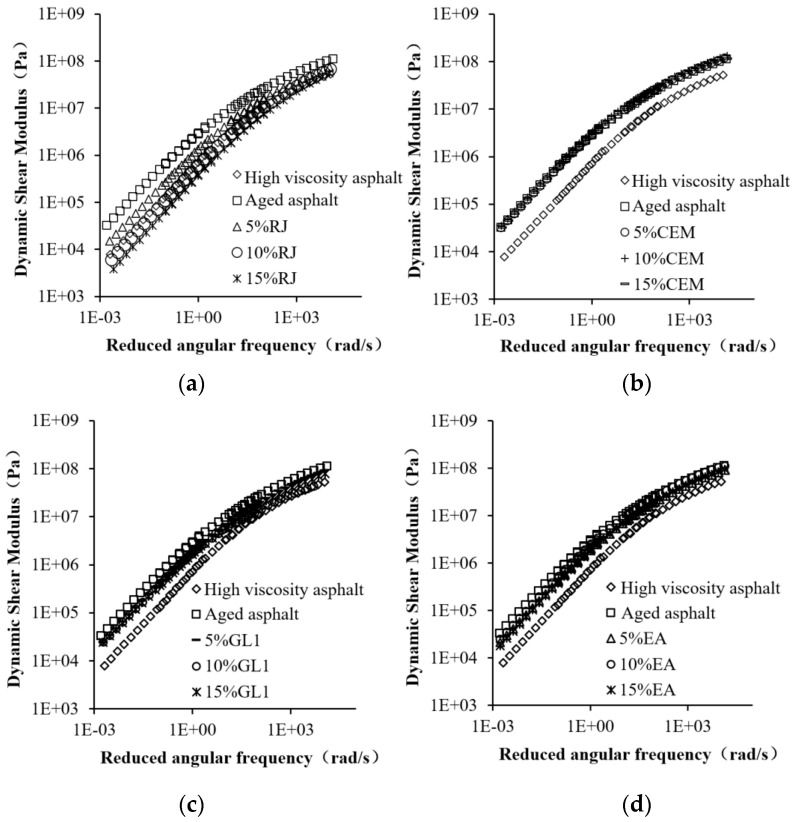
Dynamic shear modulus master curves of different asphalt materials: (**a**) RJ; (**b**) CEM; (**c**) GL1; and (**d**) EA.

In actual service conditions, asphalt pavement experiences complex axial-load loading. Therefore, a smaller *J_nr-diff_* value in asphalt material indicates lower stress sensitivity, which ultimately contributes to enhancing the service life of asphalt pavement. Figure 11 provides a summary of the *J_nr-diff_* values for different types of asphalt. It was observed that the *J_nr-diff_* value of the CEM and GL1 asphalt materials is the smallest and it is largely independent of the CEM and GL1 doping. This also indicates that both CEM and GL1 can reduce the stress sensitivity of asphalt. The *J_nr-diff_* value of RJ and EA asphalt is relatively large, which indicates that the stress sensitivity of these two is higher. However, it is important to note that the stress sensitivity of asphalt increases with a higher RJ content, while it decreases with a higher EA content. Furthermore, the addition of maintenance materials to the asphalt results in a smaller *J_nr-diff_* value compared to high-viscosity modified asphalt. This observation suggests that the four types of maintenance materials effectively enhance the stress sensitivity of the asphalt.

### 4.3. Frequency Sweep Test Results

In this section, the nonfunctional form of the time–temperature displacement factor and the CAM model are used to construct the master curve for plastic materials. Table 3 summarizes the fitting parameters of the CAM model for different materials. The dynamic shear modulus curves of different preventive materials after fitting are shown in Figure 11. As observed, the dynamic shear modulus of high-viscosity asphalt throughout the frequency domain after aging, and the modulus difference before and after aging, are especially obvious under low-frequency loading. Along with the addition of preventive maintenance materials, the mechanical properties of aged asphalt were improved to different degrees. It can be seen that RJ showed an excellent improvement effect at the appropriate content (up to 10%). As the material content increases, the dynamic shear modulus gradually decreases and even drops below the base specification of high-viscosity asphalt at 15% RJ content. This suggests that excess RJ can cause negative effects. Compared with aged asphalt, the dynamic shear modulus of asphalt was almost unchanged after adding different dosages of CEM, indicating that the CEM materials could not improve the viscoelastic behavior of aging asphalt. In addition, GL1 and EA had similar improvement effects, although the asphalt modulus was reduced to some extent at a low addition content, but the effect was not significant. The difference between the two materials is that the enhancement effect of GL1 is almost independent of the content, whereas raising the EA content is more favorable for the improvement of aged asphalt binder.

**Figure 12:**
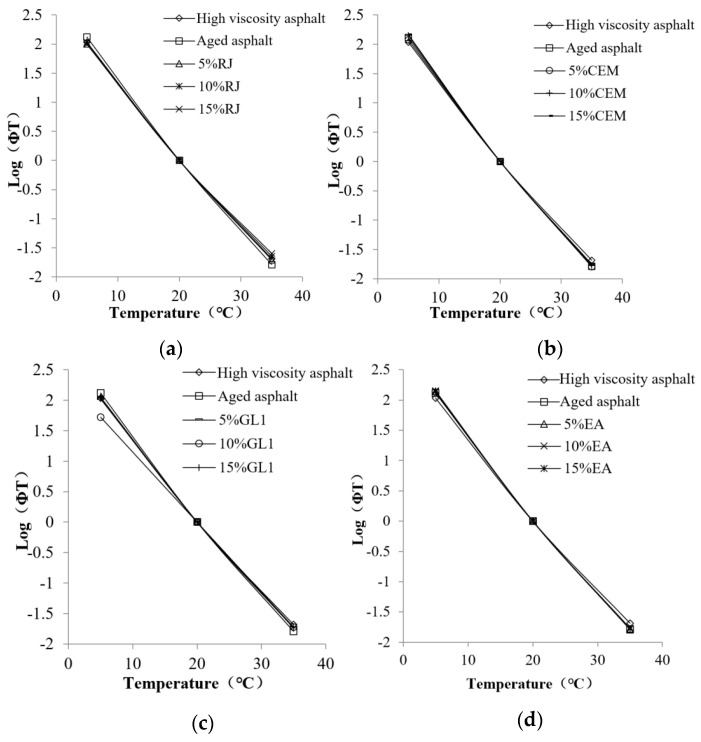
Temperature shift factor of different asphalt materials: (**a**) RJ; (**b**) CEM; (**c**) GL1; and (**d**) EA.

The fitting results of the temperature shift factor with 20 °C as the reference temperature are given in Figure 12. The temperature shift factor of the aged high-viscosity asphalt is basically the same as that of the original one. In addition, the temperature shift factor remains essentially unchanged after adding the preventive maintenance materials. The results indicate that asphalt aging and preventive maintenance materials have little effect on the “temperature offset factor” obtained from the asphalt dynamic modulus master curve fitting process. Moreover, the viscoelastic rheological temperature sensitivity of high-viscosity asphalt is only related to its constitutive properties and is independent of its aging and regeneration.

### 4.4. Accelerated Fatigue Test

**Figure 13:**
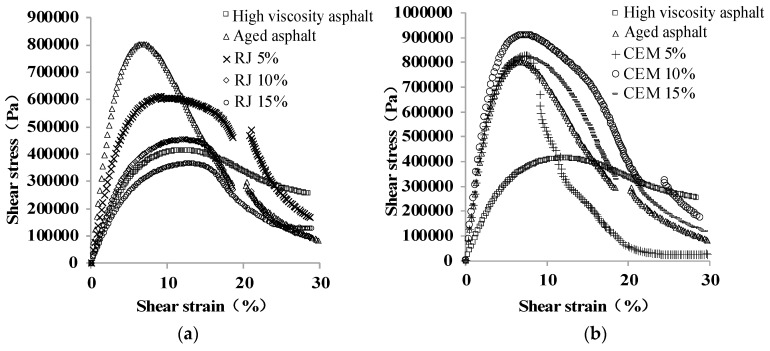

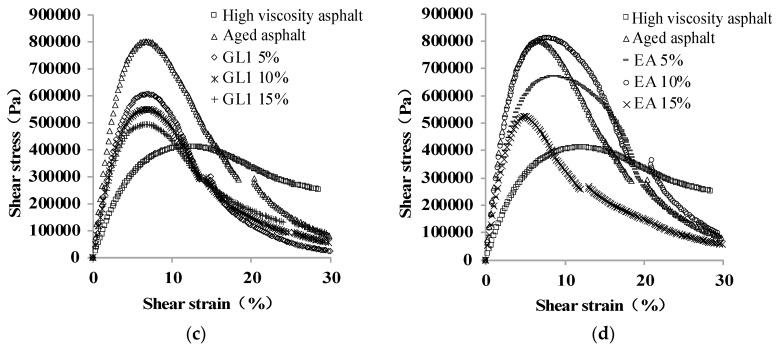
Stress–strain curves of different asphalt materials at 20 °C: (**a**) RJ; (**b**) CEM; (**c**) GL1; and (**d**) EA.

The yield stress–strain curves for high–viscosity asphalt, aged asphalt and new asphalt with various preventive maintenance materials are shown in Figure 13. Asphalt material undergoes elastic and plastic deformation sequentially under continuous loading. After the load reaches a certain limit, the material yields, and the resulting stresses and strains become yield stresses and strains. Greater the material yield stress indicates greater resistance to deformation, while higher the yield strain means that the material is more elastic.

**Figure 14:**
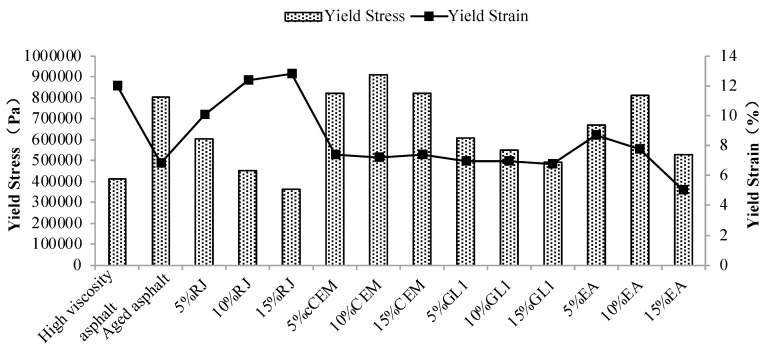
Yield stress and strain of different asphalt materials at 20 °C.

To facilitate comparison, the yield stresses and strains of different materials are extracted and displayed as shown in Figure 14. As observed, the aging effect increases the yield stress and decreases the yield strain of the asphalt, indicating that the elastic component of the asphalt decreases and the material hardens. Compared to aging asphalt, the yield stress of RJ-improved asphalt decreased while the yield strain increased. This suggests that RJ material is helpful in improving the elastic components of aging asphalt and restoring its mechanical properties. The yield stresses and strains of CEM-improved asphalt were close to those of aged asphalt, and the yield values remained essentially unchanged with changes in the CEM content. Therefore, CEM exhibited the least ability to improve the fatigue performance of aged asphalt among the four preventive maintenance materials. Although the yield strain of GL1-improved asphalt is essentially comparable to that of aged asphalt, the yield stress is significantly lower than the yield stress and decreases slightly with increasing material content. Since GL1 is an adhesive material with poor fatigue resistance, it can improve the yielding action of asphalt. The improvement effect of EA is not stable relative to aged asphalt. As the EA content increases, the yield stress first increases and then decreases, while the yield strain continues to decline. This means that although EA affects the fatigue resistance of aged asphalt to a certain extent, the effect is small and unstable. Consequently, it can be inferred that CEM and GL1 exhibit the least favorable impact on enhancing the fatigue life of aging asphalt, while RJ demonstrates the most significant effect in improving the fatigue life.

### 4.5. BBR Test Results

The results of the low-temperature PG ratings of several materials are shown in Table 4. As observed, the low-temperature rating of RJ-improved asphalt as well as the high-content GL1-improved asphalt (10% and 15%) is less than or equal to −18 °C, while that of the other asphalt materials is −12 °C. This means that RJ and high-content GL1 are beneficial in improving low-temperature performance.

**Figure 15:**
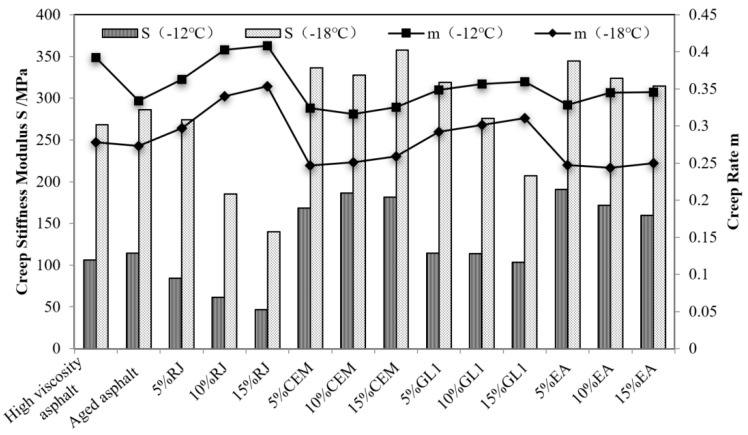
Creep stiffness modulus and creep rate of different asphalt materials at −12 °C and −18 °C.

The creep stiffness modulus and creep rate of the different materials in the BBR test were extracted and the comparison results are shown in Figure 15. As observed, after PAV aging, the elastic composition and low-temperature cracking resistance of high-viscosity asphalt decreased. After the addition of RJ material, the creep stiffness modulus of aged asphalt decreased, the creep rate increased, and the low-temperature cracking resistance was significantly improved. At a low content, the effect of GL1 on the low-temperature properties of aged asphalt was small, but with GL1 reaching 10%, the creep stiffness modulus and creep rate of aged asphalt were obviously improved under different low-temperature conditions. This indicates that the improvement effect of GL1 has certain requirements in terms of the content. In addition, the low-temperature performance of the asphalt improved by EA and CEM was significantly reduced, indicating that the aging asphalt was not only not improved but also degraded after the addition of these two materials.

## 5. Conclusions

In this study, RJ, CEM, polymerization reaction and EA were selected to improve aged high-viscosity asphalt pavements. The frequency scanning, temperature scanning, MSCR, LAS, and BBR tests were used to evaluate the temperature sensitivity, rheological properties, and fatigue performance of four preventive maintenance materials. Based on the analysis, the main conclusions are as follows:

(1) Rejuvenator (RJ), with its oily component, significantly enhances the temperature sensitivity of aged asphalt; however, the content should not exceed 10% to maintain adequate rutting resistance. Conversely, CEM, GL, and EA have minimal effects on the temperature sensitivity of aged asphalt and do not notably improve the high-temperature performance of aged asphalt binder.

(2) The addition of RJ substantially reduces the creep of aged asphalt, with the improvement becoming more significant as the RJ content increases. While increasing the EA content partially restores the performance of aged asphalt, the recovery ability is limited. CEM and GL1 show inefficacy in enhancing the deformation resistance of aged asphalt, and their effectiveness remains unaffected by content variations.

(3) RJ exhibits the most significant improvement in the aging asphalt modulus at contents below 10%; however, excessive amounts of RJ can have adverse effects. CEM does not enhance the viscoelastic behavior of aged asphalt. Notably, GL1 and EA demonstrate similar improvement effects, with GL1’s enhancement being content-independent, while a higher EA content proves more beneficial in improving the aging effect of the asphalt binder.

(4) Asphalt aging and preventive maintenance materials do not impact the “temperature offset factor” in the dynamic modulus master curve. Additionally, the viscoelastic rheological temperature sensitivity of high-viscosity asphalt is solely dependent on its constitutive properties and remains unaffected by aging and regeneration processes.

(5) RJ effectively enhances the fatigue properties of aged asphalt by introducing elastic components to restore its elastic properties. The yield point of CEM-improved asphalt closely aligns with that of aged asphalt and does not significantly affect fatigue resistance. Although GL1 and EA demonstrate some improvement potential, the effect is minor and inconsistent.

(6) Regarding the low-temperature cracking resistance of aging asphalt, RJ shows the most significant improvement, followed by a high content of GL1. In contrast, EA and CEM exhibit minimal improvement and introduce unfavorable factors in this aspect.

## Figures and Tables

**Table 1:** Property indexes of PG 58-22 virgin asphalt.

Index	Unit	Test Value
Needle penetration (25 °C, 100 g, 5 s)	0.1 mm	64.4
Softening point (ring and ball method)	°C	47.8
Ductility (5 °C, 5 cm/min)	cm	0
Ductility (15 °C, 5 cm/min)	cm	>100
Density	g/cm^3^	1.037
135 °C Brookfield viscosity	Pa·s	1.40

**Table 2:** Property indexes of high-viscosity additive.

Index	Unit	Test Value
Appearance	-	Granular, uniform and full-bodied
Single particle quality	g	0.022
Density	g/cm^3^	0.978
Melt index	g/10 min	9.861

**Table 3:** Fitting parameters and temperature shift factors.

Asphalt Type	*G_g_*	*f_c_*	*k*	*m*	*C*_1_	*C*_2_
High-viscosity asphalt	1 × 10^9^	9.5777177	0.1348616	1.1347736	19.526424	158.93497
Aged asphalt	1 × 10^9^	0.0680324	0.1257191	1.3675772	23.077429	178.37491
5% RJ	1 × 10^9^	0.8385586	0.1286189	1.2480674	23.129892	188.08075
10% RJ	1 × 10^9^	190.13767	0.159361	0.9850111	17.264238	142.38879
15% RJ	1 × 10^9^	1046.3393	0.1715793	0.9287384	15.647855	132.33103
5% CEM	1 × 10^9^	0.6646067	0.1356676	1.1980673	26.229616	204.47641
10% CEM	1 × 10^9^	0.2216911	0.1342907	1.2934787	20.500746	158.09426
15% CEM	1 × 10^9^	0.1536139	0.1304719	1.329253	18.638511	144.18936
5% GL1	1 × 10^9^	1.7735001	0.1326579	1.1387232	24.148824	193.93438
10% GL1	1 × 10^9^	3.096 × 10^−6^	0.0967924	2.4196246	61713.519	538018.29
15% GL1	1 × 10^9^	3.8443497	0.1304975	1.0669395	22.895091	182.68134
5% EA	1 × 10^9^	0.3045339	0.1260049	1.275951	20.608818	159.08111
10% EA	1 × 10^9^	0.7547354	0.1364739	1.2272653	20.031473	154.76634
15% EA	1 × 10^9^	0.641712	0.131139	1.2483411	21.2305	164.01913

**Table 4:** Low-temperature grades of different asphalt materials.

Asphalt Type	Low-Temperature Grade
High-viscosity asphalt	−22 °C
Aged asphalt	−22 °C
5% RJ	≤−28 °C
10% RJ	≤−28 °C
15% RJ	≤−28 °C
5% CEM	−22 °C
10% CEM	−22 °C
15% CEM	−22 °C
5% GL1	−22 °C
10% GL1	≤−28 °C
15% GL1	≤−28 °C
5% EA	−22 °C
10% EA	−22 °C
15% EA	−22 °C

## Data Availability

Some or all data or models that support the findings of this study are available from the corresponding author upon reasonable request.
